# Use of Artificial Intelligence in Psychiatric Research and Practice: A Qualitative Interview Study with Experts from Psychiatry, Computer Science and Philosophy in Germany

**DOI:** 10.1192/j.eurpsy.2025.10075

**Published:** 2025-08-04

**Authors:** Eike Buhr, Marc Fischer, Olga Biernetzky, Stefan Teipel, Oliver Gruber, Mark Schweda

**Affiliations:** 1 Ethics in Medicine, Carl von Ossietzky University of Oldenburg, Oldenburg, Germany; 2 Section for Experimental Psychopathology and Neuroimaging, Ruprecht Karl University of Heidelberg, Heidelberg, Germany; 3 Department of Psychosomatic Medicine, University Medicine Rostock, Rostock, Germany; 4 German Center for Neurodegenerative Diseases (DZNE), Rostock, Germany

**Keywords:** artificial intelligence, dementia, depression, ethics, qualitative interview study

## Abstract

**Background:**

The use of artificial intelligence (AI) in psychiatry holds promise for diagnosis, therapy, and the categorization of mental disorders. At the same time, it raises significant theoretical and ethical concerns. The debate appears polarized, with proponents and critics seemingly irreconcilably opposed. On the one hand, AI is heralded as a transformative force poised to revolutionize psychiatric research and practice. On the other hand, it is depicted as a harbinger of dehumanization. To better understand this dichotomy, it is essential to identify and critically examine the underlying arguments. To what extent does the use of AI challenge the theoretical assumptions of psychiatric diagnostics? What implications does it have for patient care, and how does it influence the professional self-concept of psychiatrists?

**Methods:**

To explore these questions, we conducted 15 semi-structured interviews with experts from psychiatry, computer science, and philosophy. The findings were analyzed using a structuring qualitative content analysis.

**Results:**

The analysis focuses on the significance of AI for psychiatric diagnosis and care, as well as on its implications for the identity of psychiatry. We identified different lines of argument suggesting that expert views on AI in psychiatry hinge on the types of data considered relevant and on whether core human capacities in diagnosis and treatment are viewed as replicable by AI.

**Conclusions:**

The results provide a mapping of diverse perspectives, offering a basis for more detailed analysis of theoretical and ethical issues of AI in psychiatry, as well as for the adaptation of psychiatric education.

## Introduction

The successful use of artificial intelligence (AI) in medical fields, such as oncology, radiology, and ophthalmology [1], cannot simply be transferred to applications in psychiatry. Unlike these fields, where diagnoses can often be made based on quantifiable biological or imaging markers, psychiatry deals with complex mental disorders that encompass biological, psychological, and social aspects [2]. While many approaches place great emphasis on the biological perspective [3, 4], framing mental disorders as brain diseases [5], others highlight the role of the mind and the subjective experience of the patient [6]. These ambiguities complicate the integration of AI tools and raise significant theoretical and ethical questions: How do AI-based systems influence our understanding of psychiatry and the diagnosis of mental disorders? What impact do AI-driven approaches have on patient care, the role of psychiatrists, and the doctor–patient relationship? Despite the growing relevance of these questions, the perspective of experts on the integration of AI into psychiatry – particularly in Germany – has, with few exceptions [7], received little systematic attention.

To explore these issues, we conducted semi-structured interviews on AI in psychiatry with 15 experts from the fields of psychiatry, computer science, and philosophy in Germany. The primary objective was to evaluate technical aspects, fundamental theoretical and ethical questions, and attitudes of psychiatrists, providing a deeper understanding of the underlying arguments. Qualitative interviews are particularly suitable for this purpose as they allow for assessing the background assumptions and considerations of the participants in more detail. The psychiatrists interviewed are leading researchers in the fields of dementia and depressive disorders. These diseases are particularly suitable examples as the scientific understanding of their pathomechanisms covers the range between a primarily biological concept in the case of dementia and a biopsychosocial concept in the context of depression [8, 9]. In addition to exploring the feasibility and desirability of using AI-based methods for psychiatric diagnosis, we specifically examined attitudes toward the technical aspects and ethical implications of integrating AI into psychiatry. To this end, we also included computer scientists and philosophers in our study to complement clinical expertise with technical and normative perspectives. In particular, the inclusion of philosophers enabled us to explore ethical and epistemological issues that are often not explicitly addressed in clinical or technical contexts. Overall, this approach enabled a comprehensive overview of the current state of research from the perspective of psychiatry, computer science, and philosophy, also highlighting theoretically and ethically significant issues and challenges.

## Study design and methods

To explore expert perspectives on AI in psychiatry and provide a detailed analysis of their arguments [10], we employed a qualitative research design using semi-structured interviews. Participants were purposively selected based on their expertise in psychiatry, computer science, and philosophy (specializing in ethics or epistemology). Inclusion criteria consisted of demonstrated experience and professional recognition in their respective fields, as reflected, for instance, in senior academic positions.

A total of 15 experts participated in the study: 5 psychiatrists specializing in dementia, 5 psychiatrists with a focus on depression, 1 computer scientist, and 4 philosophers (1 originally trained in psychiatry). All participants held full professorships, except for one expert who held a temporary professorial position. Participants were identified based on their contributions to relevant scholarly and clinical debates, alignment with the research focus, or through the professional networks of the research team.

Data were collected through semi-structured interviews conducted between September 2023 and September 2024. The interview guideline encompassed open questions regarding understandings of AI, its role in research work, and its perceived impact on psychiatric diagnosis, therapy, and the character of psychiatry as a discipline. Moreover, we asked for the potential impact of AI on patient care, the professional identity of psychiatrists, and the doctor–patient relationship. In this study, we focus on AI’s influence on diagnosis, patient care, and the role of the psychiatrist.

All interviews were conducted in German via video conferencing platforms and lasted between 30 and 90 min. They were recorded and transcribed for analysis. The study design was approved by the ethics committee of the Medical Faculty of the University of Rostock on June 22, 2023 (A 2023-0110). All participants provided informed consent before the interviews.

The interviews were audio-recorded, transcribed, coded using MAXQDA®, and interpreted using qualitative content analysis [11]. Due to the exploratory character of the study, a *structuring* content analysis was deemed most suitable [11]. The coding guideline contained descriptive codes in accordance with the interview questions and was tested via “subjective assessment” [12].

The analysis was conducted in several steps: First, we developed the main categories based on our research questions and the interview guideline. Next, we tested the coding guideline to ensure intercoder agreement and then applied this refined guideline to code the data [12]. Finally, we organized the data according to the main categories and further structured these categories in alignment with our research questions. The results of our analysis include the most prevalent themes as well as the underlying arguments.

## Results

This section synthesizes the findings from the expert interviews. The subsections cover potential applications of AI for psychiatric diagnosis, its influence on patient care, and the role of the psychiatrist. They include both theoretical and ethical considerations.

### Expert perspectives on potential AI applications for psychiatric diagnosis

The interviewed experts identified various possible applications for AI as a diagnostic tool in psychiatry. Their perspectives also reflect divergent conceptual understandings of psychiatry as a discipline. One line of argument was based on the idea that “if we had a huge data set about a person, then I might have a lot more information that could also lead to a better understanding” (Philosopher with a focus on philosophy of psychiatry).

The experts pointed to various data sources that could serve this purpose, ranging from structural and functional imaging data to data on facial expression or behavior. With reference to a digital pen that helps with dementia diagnosis, one computer scientist explained that the benefits of such applications rely on both the amount and type of data available:

“What is crucial here is that the AI not only automatically evaluates these tests, but that there are also additional signals coming through this digital pen. Parameters that cannot otherwise be detected by humans. […] And perhaps it identifies certain characteristics that the doctor hadn’t considered. And that is a real added value, which you get when you capture such tests with sensors and evaluate them automatically” (Computer Scientist with a focus on medical applications).

This statement suggested that the use of AI in psychiatric diagnostics initially involves gathering more data about the patient but also uncovering information that is beyond the reach of human perception. Importantly, this was tied to the hope that AI could lead to more precise and reliable psychiatric diagnoses, as one expert explained:

“Because psychiatric diagnostics are so imprecise, there are, of course, many attempts to use pattern recognition in various diagnostic, imaging, or other technical diagnostic procedures to establish classifications and groupings” (Psychiatrist with a focus on dementia, 1).

As AI systems excel at recognizing patterns in large datasets, some undetected by humans, it was argued that AI could help make psychiatric diagnoses more precise, either by capturing parameters that cannot be detected by humans or have not been considered by them, or because AI has access to and oversight over a larger amount of data. This is not limited to the field of dementia, where the understanding of the pathomechanistic concept is more advanced. For example, another participant expressed hope that AI approaches could also help identify biomarkers “in the context of depression, […] in the development of biomarkers, psychiatry can definitely change. I’m very optimistic about that” (Psychiatrist with a focus on depressive disorders, 1).

Alongside neurobiological data as the basis for identifying biomarkers, additional sources of data that could be valuable for diagnosing mental disorders were also highlighted – for example, in terms of “facial expressions, gestures, voice modulation, motor skills. How does someone move? […] Step count? Whatever” (Psychiatrist with a focus on depressive disorders, 2). In clinical practice, mental disorders are commonly diagnosed using questionnaires that focus on behavior, perception, emotions, or thoughts. However, behavioral markers have also been discussed for some time, such as movement patterns. The following expert highlighted the potential role of AI systems in capturing and analyzing these markers and explained:

“when we see depressive patients […] walk in through the door, I can […] make the diagnosis in 80% of cases right away. If they even open their mouth, maybe 90%, without them saying anything substantive. And that […] is some kind of pattern that one develops with a certain amount of experience in psychiatry and that could certainly also be displayed technically” (Psychiatrist with a focus on depressive disorders, 1).

The expert expresses hope that his clinical experience and intuitive judgment in diagnosing depression through gait and speech patterns might be programmed into AI systems. Thus, this experience and judgment do not seem to be regarded as uniquely human competences but rather as something that could also be translated into technical functions. The quoted expert envisioned a broad range of possible applications and data sources, indicating that the application of AI in psychiatry might not be confined to neurobiological data alone.

The expert statements highlight various potential applications of AI in diagnostic practices. Their assessments of AI’s role in diagnosis vary between a supplement to human expertise, a means of replicating and standardizing it, or an equivalent or even superior approach. Notably, the envisioned potential applications differ primarily regarding the types of data considered relevant for AI-driven systems.

### Expert perspectives on AI’s influence on patient care

Regarding AI’s influence on patient care, we could identify two lines of argument. The first one expresses concern about the expansion of psychiatric diagnoses and the risk of pathologization and dismissal of the patient’s subjective experience. This argument highlights AI’s inability to consider contextual factors when diagnosing mental disorders, questioning whether labeling every change or deviance in the data as a symptom of a mental disorder is beneficial. The second line of argument stresses the importance of distinct human abilities that are essential to psychiatric practice and cannot be replaced by AI. These include considering the patient’s subjective perspective, communicating diagnoses in a sensitive way, and showing empathy for the patient’s condition.

One expert argued that the reliance on neurobiological data could dismiss the patient’s subjective perspective, potentially excluding patients from psychiatric care “if these criteria defined at some point are not met by individual patients and they are still ill” (Psychiatrist with a focus on depressive disorders, 1). At the same time, when asked whether AI implementation could simplify diagnosis, another expert expressed concern that AI lacks the necessary sensitivity, thus leading to an expansion of psychiatric diagnoses:

“DSM-5 is already heading in the direction where we say you have more or less a personality disorder, for example. I find this trend will increase in medicine, and I have a major concern that, in the end, there will be no normal people left, because anyone who stands out in some dimension will be classified, and whether this seriously benefits humanity is one question, and whether it benefits the individual person is even more of a question. […] AI diagnosis does not consider this” (Psychiatrist with a focus on depressive disorders, 2).

While AI could support more comprehensive diagnostics, the cited expert also expressed concern about an expansion of psychiatric diagnoses, as this might lead to recognizing nearly every tested individual as having some form of mental disorder. Although AI can process a wider range of data sources, the cited expert feared that it cannot make contextual judgments about the impact of communicating a diagnosis to the patient.

However, the growing scope of psychiatric diagnoses also presents challenges for neurobiological evaluations – for example, in terms of preventive diagnostics. Thus, the aforementioned expert warned that “if you send all sorts of healthy people into early detection programs, something unusual always comes up, and we end up defining actually healthy, symptom-free people as sick, […] who aren’t even treated because treatment doesn’t make any sense. But the people are then extremely afraid” (Psychiatrist with a focus on depressive disorders, 2).

Overall, the identified concerns apply to both behavioral and (neuro)biological markers. At the same time, experts emphasized genuinely human skills that are crucial for psychiatry and cannot be replaced by AI, such as sensitive communication and consideration of the patient’s individual perspective. This indicates that positions on the use of AI in psychiatry mainly depend on the belief in certain irreducibly human abilities required for diagnosis and therapy.

### Expert perspectives on AI’s influence on the role of psychiatrists

Regarding impacts on the role of the psychiatrist, we identified three lines of argument. The first challenges the possibility for AI to capture every factor that is relevant for a mental disorder. For example, one expert argued that AI might not be able to incorporate every aspect of the human condition into data:

“AI-supported solutions can only ever process the data […] that is provided to them […], and there are always many other data that also […] make up an individual person […] that can be sensed and recognized through […] analogies, feelings like empathy, and whose meaning […] is captured in the interaction with the patient which, however, can never be included in the calculations of this AI system” (Psychiatrist with a focus on dementia, 2).

The expert argued that certain human competencies are necessary to capture the condition of the patient and cannot be technically replicated in AI systems. Here, the interaction between the patient and psychiatrist is deemed necessary for perceiving aspects that make up the individual and their mental disorder.

However, experts also acknowledged that AI has the potential to deliver more accurate results than even experienced psychiatrists:

“That will then be measured and validated against clinical assessment […]. And I’m very curious to see who will be more accurate […]. I can well imagine that AI-supported systems, which don’t have the individual limitations that every doctor has and preferences […] which are more emotional […] and not defined by data, that such algorithms could actually approach these issues much better” (Psychiatrist with a focus on dementia, 2).

If AI applications are in fact more accurate than human practitioners, one expert argued that we should “consider how to prevent a nonsensical human intervention, when the AI decision is better” (Psychiatrist with a focus on depressive disorders, 3). In contrast to the argument that psychiatry depends on inherently human abilities like empathy, this second line of argument relies on the assumption that emotions and individual preferences introduce bias, suggesting a different role of the psychiatrist in relation to the AI.

According to the third line of argument, the implementation of AI in psychiatry runs the risk of reducing disease entities to measurable data, as these are easier to integrate into AI, as exemplified by the following quote:

“So, this can so to say lead to the reduction of disease patterns to more easily measurable parameters […]. You can, in a sense, represent certain phenotypes that, through the linking of large datasets, may appear justified or validated in correlation, […] which in reality may not exist at all. This is often discussed […] under psychiatrization of everyday phenomena” (Philosopher and physician with a focus on AI and medical ethics).

The expert pointed to the fundamental issue of data bias, that is, systematic distortions within datasets as a consequence of simplifying disease patterns in terms of measurable data. Here, the quoted expert articulated the concern that the use of AI may encourage a simplification of disease models, favoring more measurable and operationalizable terms. As a result, certain symptoms may be misrepresented, leading to an exaggerated perception of their correlation with specific outcomes. This not only affects behavioral data or digital markers but also neurobiological data.

## Discussion

In our analysis of attitudes and perspectives on AI in psychiatry, we identified different lines of argument that also reflect diverse perspectives on psychiatry as a discipline. The juxtaposition of these perspectives highlights a fundamental challenge in applying AI to psychiatry: the lack of consensus on the nature of mental disorders and their classification [13–15]. However, our results indicate that the theoretical perspective reflected in the respective statements does not directly determine specific attitudes toward the use of AI. Instead, it shapes which type of data are considered suitable for AI-driven analysis.

Regarding *potential applications* of AI, one line of argument highlights the potential of neurobiological and genetic data and states that AI could aid in developing new classification systems for mental disorders, addressing challenges such as transdiagnostic symptoms, complex differential diagnoses, and low interrater reliability [16, 17]. Here, AI is seen as a complement that compensates for psychiatrists’ limitations by processing large datasets and identifying previously unknown patterns. This view also has practical implications: by identifying the neurobiological foundations of mental disorders, AI could also help to reduce stigma [18]. Another perspective highlights that AI systems could also process and integrate behavioral data, voice, facial expressions, or gait. Here, AI is rather seen as a means of operationalizing psychiatrists’ expertise and standardizing psychiatric assessments. Since the data used in AI-based diagnostic applications point to the aspects deemed constitutive of mental disorders, the experts’ statements also highlight the influence of AI on psychiatric nosology.

On an *ethical level*, we found a range of perspectives regarding AI’s influence on psychiatric patient care. One touches upon the issue of dealing with discrepancies between AI and patient assessments of mental health [18, 19]. Proponents of this stance suggest that AI use could contribute to the expansion of psychiatric diagnoses, influencing societal attitudes toward mental disorders. In fact, many symptoms of mental disorders are defined in broad terms, meaning that most people experience them to some extent at various points in their lives [20]. Furthermore, digital markers have not yet been validated to a degree that allows for unequivocal diagnoses. Critics highlight that studies in this area often overestimate the validity and reliability of such markers [21].

The second line of argument holds that certain genuinely human abilities are essential to psychiatric practice and unachievable for AI. The idea that qualities like empathy are uniquely human, coupled with concerns about replacing human psychotherapists, is a recurring theme in the ethical literature [22]. However, empirical studies show that individuals sometimes perceive AI-generated responses to health issues as more empathetic than those of human physicians [23]. This raises the fundamental question of the uniquely human status of certain abilities, and of their definition, underlying mechanisms, and development. Moreover, some people argue that some individuals may prefer interacting with an AI or chatbot due to feelings of shame or discomfort when discussing their mental health with another person [24].

Regarding the influence of AI on the *role of psychiatrists*, we could identify three lines of argument. The first expresses concern that approaches advocating for neurobiological data in AI-driven psychiatric diagnostics run the risk of dehumanization or the oversimplification of mental health conditions by emphasizing biological data, potentially excluding patients from psychiatric care, and neglecting the role of a trusting doctor–patient relationship. This issue is also discussed in ethical and theoretical literature emphasizing non-measurable dimensions, such as consciousness or the self [6, 18, 19].

Another line of reasoning sees human characteristics, such as emotionality, as a limitation of judgment that can be compensated for through AI. This line raises questions about the accuracy of AI in relation to that of experienced psychiatrists and the potential obligation to follow AI-based recommendations. This brings up issues of accountability, such as the degree of accuracy an AI system must reach before its recommendations become obligatory, and whether psychiatrists could be held responsible for disregarding AI suggestions [25]. This would impose additional demands on psychiatrists, such as a deeper understanding of the validity of AI results and the underlying data.

The third line warns of a reliance on data that are easily measurable and computable and risks reducing the complexity of mental disorders. This could result in correlations where certain factors appear, particularly risky or pathological, even though their actual significance is more limited. The issue of data bias is already widely discussed in the context of AI in medicine, a fundamental challenge being the opacity of AI systems [26]. These concerns underscore the critical role of epistemological questions, especially regarding AI accuracy and explainability, when addressing ethical issues [27]. Such biases also affect the role of psychiatrists, raising the question of whether psychiatric training should incorporate education on AI systems to enable clinicians to recognize bias and assess its impact.

Overall, the findings of our study suggest that disciplinary backgrounds might influence how experts reason about the use of AI in psychiatry. For instance, the computer scientist interviewed emphasized technical feasibility and adopted a predominantly utilitarian cost–benefit perspective in ethical considerations. In contrast, the philosophers focused on foundational epistemological and normative questions. The psychiatrists, by comparison, grounded their arguments more directly in concrete clinical scenarios and practical concerns.

Our study has several limitations that need to be considered. Due to the small sample size, the results are not generalizable. Furthermore, the qualitative analysis of the interview material also involves a certain extent of subjective interpretation. Finally, the study reflects expert perspectives from Germany that may differ from those in other national contexts. In the underexplored field of stakeholder perspectives on AI in psychiatry, however, our exploratory approach still provides first hypotheses that can be further tested in larger quantitative surveys and international comparisons.

## Conclusion

This study advances the empirical investigation into AI’s role in psychiatric research and practice. Its results suggest that the key difference in arguments in favor of or against the implementation of AI in psychiatry lies in the types of data considered relevant for psychiatric application, and in beliefs regarding the existence of genuinely and irreplaceably human skills and their role in diagnosing and treating mental disorders. In addition, we can complement the theoretical perspective presented in previous expert interview studies [7], with an explicitly ethical perspective.

Our findings emphasize the need for further theoretical analysis regarding the epistemic challenges of different types of data when processed by AI. Moreover, they point to the need to explore the notion of distinctly human abilities, their role in the diagnosis and treatment of mental disorders, and their cultivation in psychiatric education. This also suggests that the training of psychiatrists should focus on developing skills that strengthen the doctor–patient relationship. At the same time, it is essential to develop concepts that integrate digital competencies into medical education, both for doctors in general and for psychiatrists in particular. This integration should prioritize not just practical skills but, more importantly, reflective skills that enable a critical evaluation of AI in psychiatry.

## Data Availability

The data that support the findings of this study are available from the corresponding author, EB, upon reasonable request.
